# The protective effect of Er-Xian decoction against myocardial injury in menopausal rat model

**DOI:** 10.1186/s12906-018-2311-9

**Published:** 2018-09-03

**Authors:** Zhiguo Zhang, Lihua Xiang, Lanping Zhao, Hong Jiao, Zhen Wang, Yubo Li, Yanjing Chen

**Affiliations:** 1 Institute of Basic Theory, China Academy of Chinese Medical Sciences, Beijing, 100700 China; 2 Basic Medical College, Hebei North University, Zhangjiakou, 075000 Hebei China

**Keywords:** Er-Xian decoction, Cardioprotective effect, Cardiomyopathy, Menopause, Ovariectomy

## Abstract

**Background:**

Er-Xian decoction (EXD), a formula of Chinese medicine, is often used to treat menopausal syndrome in China. The aim of the present study was to explore the potential cardioprotective mechanism of EXD against myocardial injury in an ovariectomy-induced menopausal rat model.

**Methods:**

We divided the female Wistar rats into ovariectomy group and sham operation group (SHAM group). The ovariectomized (OVX) rats received treatment of vehicle (OVX group), EXD (EXD group) or 17β-estradiol (E2 group). After 12-week of treatment, the level of estradiol in serum was detected using an electrochemiluminescence immunoassay, and electrophysiologic changes in myocardial action potentials (AP) were evaluated using intracellular microelectrode technique. Changes in the histopathology of the left ventricle and the ultrastructure of the cardiomyocytes were observed by hematoxylin and eosin (HE) staining and transmission electronmicroscopy to assess myocardial injury. Microarrays were applied for the evaluation of gene expression profiles in ventricular muscle of the OVX and EXD rats. Further pathway analyses of the differential expression genes were carried out using the Kyoto Encyclopedia of Genes and Genomes (KEGG). And real-time quantitative RT-PCR (qRT-PCR) was used for verification of the key findings.

**Results:**

The results from electrophysiological and histomorphological observations demonstrated that EXD had a substantial myocardial protective effect. The EXD-treated rats, in comparison with the OVX rats, demonstrated up-regulated expression of 28 genes yet down-regulated expression of 157 genes in the ventricular muscle. The qRT-PCR assay validated all selected differential expression genes. The KEGG pathway analysis showed that the down-regulated genes were relevant to cardiomyopathy and myocardial contractility. EXD could decrease the mRNA expressions of cardiac myosin (*Myh7*, *Myl2*) and integrin (*Itgb5*) in the ventricular myocardium.

**Conclusion:**

EXD had a protective effect against myocardial injury in OVX rats, and this cardioprotective effect may be associated with modulation of the expression of cardiac myosin or integrin at the mRNA level.

**Electronic supplementary material:**

The online version of this article (10.1186/s12906-018-2311-9) contains supplementary material, which is available to authorized users.

## Background

During the menopausal period, many physiologic and pathologic changes occur in this crucial stage for women because hormonal changes significantly influence several systems and organs. The cardiovascular system also suffers from structural or functional impairment [1–3]. Several studies showed that estrogen deficiency increased cardiovascular risk factors and subsequently increased cardiovascular morbidity and mortality [4, 5], and myocardial injury can take place in asymptomatic women in postmenopause [6, 7]. It is believed that the higher risk associated with early menopause is due to longstanding deprivation of endogenous estrogen, which may influence cardiovascular risk through a variety of effects on metabolism and vascular function [8]. Though hormone therapy had a protective effect on the myocardium of menopausal women [9, 10], some randomized controlled trials have shown conflicting study results [11, 12]. In addition, HT could lead to increased incidence of breast cancer and thromboembolic disease in women [13, 14]. Therefore, an effective and safe substitutional treatment should be brought forward to decrease the risk of myocardial injury in menopausal women.

The ovariectomized (OVX) rat is a suitable animal model to mimic the estrogen withdrawal of postmenopausal women [15, 16]. Some studies had shown OVX rat could undergo myocardial injury induced by ovariectomy [17–19], and estrogen had a protective effect against this myocardial injury [20, 21]. Er-Xian Decoction (EXD), a Chinese herbal formula, has been proven to reduce the severity and frequency of hot flushes and ameliorate menopausal symptoms in perimenopausal women without serious adverse events [22]. Moreover, a study based on menopausal rat model revealed that EXD showed an estrogen-like effect in the relief of menopausal syndrome [23, 24]. Naturally, we hypothesized that EXD could have a cardioprotective effect on menopausal animal models. In this study, we explored the protective role of EXD on cardiac structure and function in a menopausal rat model induced by ovariectomy.

## Methods

### Herbal materials

*Herba Epimedii*, *Radix morindae Officinalis*, *Radix Angelicae Sinensis*, *Rhizoma Anemarrhenae*, *Cortex Phellodendri* and *Rhizoma Curculiginis* were purchased from Beijing Tongrentang (Bozhou) Prepared Slices of Chinese Crude Drugs Co., Ltd. (Beijing, China), and by a pharmacist of traditional Chinese medicine (Feng Sui) in Institute of Chinese Materia Medica, China Academy of Chinese Medical Sciences. All voucher specimens are deposited in the herbarium center of China Academy of Chinese Medical Sciences.

### Preparation of EXD

We extracted a mixture of six herbal medicines (250 g of each herbal medicine, one thousand five hundred grams in total) by decoction with 10× (*v*/*w*) distilled water at the temperature of 100 °C for 2 h. After filtration, we boiled the residue for another 1 h, and mixed the filtrates together, then preserved at 4 °C after lyophilized using a freeze drier (Labconco, Freezone). The yield of the dried extract from the initial crude materials was 10%. We dissolved the extracts to 30 and 60 mg/ml by adding water, and then administered to the rats orally with a dose of 0.1 ml/kg body weight.

### Animal studies

In this study, seventy-two virgin Sprague-Dawley rats aged six months and weighing approximately 310 ± 20.0 g were acquired from the Experimental Animal Center in National Institutes for Food and Drug Control of China (SCXK (JING) 2009–0017, Beijing, China). The protocol involving animals in this study was authorized by The Institutional Ethics Committee of China Academy of Chinese Medical Sciences (Approval number: 2015–010). The sham operation (*n* = 18, SHAM group) or bilateral OVX (*n* = 54) using a dorsal incision [25] was performed on the rats after acclimatization. The rats undergoing OVX were divided into three groups based on the treatment agents, including vehicle (OVX group), EXD (EXD group) and 17β-estradiol (E2 group). Each group included eighteen rats. Subsequently, we dissolved 17β-estradiol (Sigma-Aldrich, USA) in absolute ethanol and diluted with olive oil. The preparation was used for daily subcutaneous injection of the E2 group rats at a dose of 30 μg/kg body weight. EXD was dissolved in aqua destillata and orally force-fed to the EXD group rats at a dosage of 7.5 g raw herbs/kg body weight/day, which was calculated using the human recommended dosage proposed by the Chinese Pharmacopeia and the surface area ratio of rats and humans. The OVX and SHAM rats were orally force-fed equal quantities of aqua destillata and standardized rat food was given to all rats throughout the current study. The rats were treated with agents or aqua destillata seven days after operation and the treatment lasted until the 13th week. The body weight of each subject was measured once a week.

### Arrangement of the specimens

On the day after final treatment, xylazine (12 mg/kg) and ketamine (80 mg/kg) were injected intraperitoneally to anesthetize all animals, which were subsequently exsanguinated for sacrifice.

Half of the rats in each group (*n* = 9) were used for an isolated heart perfusion assay. For the isolated heart perfusion assay, we removed the rat hearts rapidly and excised the left ventricular papillary muscles. The arrangement process was conducted in a plexiglas chamber which allowed continuous superfusion.

The other rats in each group (*n* = 9) were used for the rest experiments. In the rest experiments, the uteri of the rats were excised and immediately weighed and the aorta abdominalis was punctured before the rats died to collect blood specimens into tubes. Then, serum was prepared by centrifugation (2000 rpm for 20 min) and preserved at − 80 °C before use to determine the level of estradiol. The left ventricle of the heart was removed. We fixed a piece of the left ventricle in 4% paraformaldehyde, after fixture we dehydrated in graded ethanol and then embedded in paraffin. Then, the paraffin-embedded tissues were cut into sections (5-μm thickness) and subjected to hematoxylin and eosin (HE) staining. Another piece of the left ventricular tissue was used for observation under a transmission electronmicroscope (TEM). The remaining left ventricular tissue was preserved at − 80 °C to conduct the real-time quantitative RT-PCR (qRT-PCR) and microarrays assays.

### Measurements of estradiol in serum

The estradiol concentration in the serum was detected using an electrochemiluminescence immunoassay (ECLIA) Assay Kit purchased from Roche Diagnostics (Mannheim, Germany) in accordance with the instructions. All measurements were performed according to the manufacturers’ protocols.

### Electrophysiologic studies

We removed the rat hearts rapidly and placed in Krebs-Henseleit buffer (oxygenated, 4 °C) for removal of the extracardiac tissue. The sample (4 mm × 4 mm) was cut from the left ventricle and then pinned on a thin silicon disc at the bottom of a perfusion chamber. Thereafter, the sample was perfused (4 mL/min) with Krebs-Henseleit solution which contained (in mM) 121 NaCl, 5 KCl, 1.2 MgSO_4_, 2 CaCl_2_, 25 NaHCO_3_, 1.2 NaHPO_4_, and 11 glucose. Finally, the sample was equilibrated with 5% CO_2_ in O_2_ at 36 ± 0.5 °C and the pH was 7.36–7.42.

The whole process was stimulated at a frequency 1 Hz by 1 ms square pulse 1.5 times threshold voltage. We recorded and analyzed transmembrane action potentials (APs) using intracellular microelectrode technique (Chengyi, China). The variables measured were resting membrane potential (RMP), AP amplitude (APA), the maximal rate of depolarization (Vmax), and AP duration at 50% and 90% (APD_50_ and APD_90_).

### Histological studies

The fixture of the heart was carried out by dipping into 10% neutral buffered formalin. Parallel to the atrioventricular groove were serial sections for 5 μm made. Standard HE staining was used for the histomorphological evaluation.

### Ultrastructure observation

At the end of reperfusion, we cut fresh myocardium tissue (1 mm^3^) off from about the middle of the apex and the ligation point, then prefixed with 2.5% glutaraldehyde and stored at 4 °C until processed. The post-fixture was accomplished using 1% osmium tetroxide. Then, after dehydration with gradient ethanol, we dipped the specimens in propylene oxide, and embedded with epoxy resin. Afterwards, we made them into ultrathin sections (0.1 μm). Finally, we stained the sections with lead-uranium and observed the changes in the ultrastructural organization by a TEM (HITACHI-S3400 N, Japan).

### Microarray data analysis

We conducted the gene microarray assay using KangChen Bio-tech (Shanghai, China). The microarrays used in the present study were whole-genome gene expression profiling chips (4 × 44 K) containing about 41,000 probes for rat genes, which were purchased from Agilent Technologies. The collection of total RNA of the left ventricular tissue from the EXD group and the OVX group as well as a DNase digestion step was accomplished with the help of the RNeasy kit (Qiagen) and TRIzol (Invitrogen) according to the instructions. We amplified and labeled the samples with the help of an Agilent Quick Amp labeling kit, then we hybridized the samples using an Agilent whole-genome oligo microarray, following RNA measurement by the NanoDrop ND-1000 and denaturing gel electrophoresis. All the processes were conducted in Agilent’s Sure-Hyb Hybridization Chambers. We scanned the processed slides with the help of the Agilent DNA microarray scanner following hybridization and washing, in accordance with the settings provided by Agilent Technologies. We extracted the text files generated out of the Agilent Feature Extraction Software. Then input them into the version 11.0 of Agilent GeneSpring Software in order to analyze deeply. The gene expression level of the OVX group was considered as normal. Fold changes (≥2.0) and t-test *p* value (≤0.05) screening were applied for the identification of differentially expressed genes.

### Pathway analysis

We used the Kyoto Encyclopedia of Genes and Genomes (KEGG) [26] for the pathway analysis to explore the potential role specific genes played in certain biological processes. We imported the differential expression genes between the EXD group and OVX group into the KEGG and the top pathways (*P* value≤0.01) associated with myocardial morphology or functions were observed.

### qRT-PCR analysis

We purified total RNA with the help of an RNeasy Mini Kit (Qiagen), then reverse-transcribed 4 μg of RNA to cDNA with the help of the Superscript First Strand synthesis system (Invitrogen). Afterwards, with the help of an ABI-7500 Sequence Detection System (Applied Biosystems), we performed qRT-PCR amplification with SYBR-green according to the instructions, which could detect PCR products in real time. Initiation of the PCR program was at 95 °C for 10s before 40 thermal cycles, then at 95 °C for 5 s and at 60 °C for 34 s. The data were analyzed in accordance with the 2^−△△Ct^ method and in each sample, the results were normalized to the expression of glyceraldehyde-3-phosphate dehydrogenase (*Gapdh*). To guarantee the purity of the amplification products, we created melting curves for each of the PCR reactions. Each of the experiments was matched with a no-template negative control.

### Statistical analysis

All the values were presented in the way of mean ± standard deviation. SPSS 13.0 (SPSS Inc.) was applied for all statistical analyses. The least significant difference (LSD) test and the analysis of variance (ANOVA) were applied for the test of differences between groups concerning the evaluated parameters. The data of all groups went through normality test by the method of Kolmogorov-Smirnov test. Statistical significance was defined as *P* value< 0.05.

## Results

### Effect of EXD on the weights of bodies and uteri

**Fig. 1:**
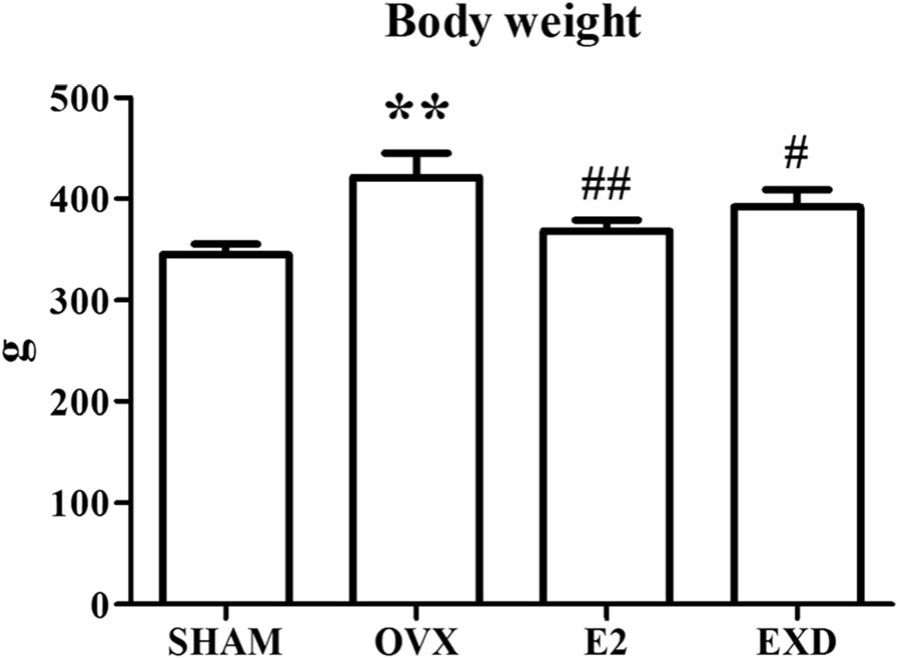
Effect of EXD on body weight after 12-week treatment. ** *P* < 0.01 versus SHAM group; ^#^ *P* < 0.05 versus OVX group; ^##^ *P* < 0.01 versus OVX group

Figure 1 shows that the SHAM rats weighted remarkably less compared with the OVX rats. Both E2 and EXD could remarkably inhibit weight gain induced by OVX during the twelve-week treatment.

**Fig. 2:**
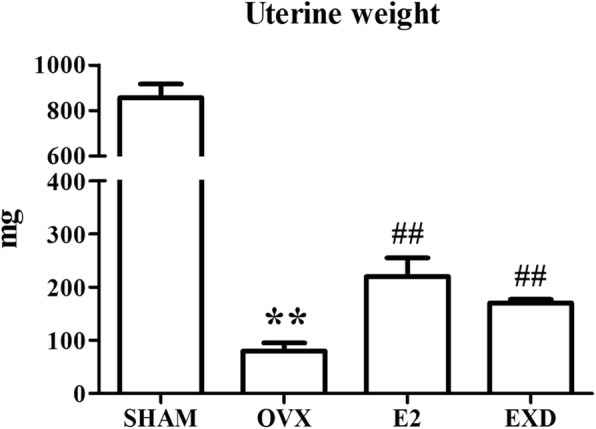
Effect of EXD on uterine weight after 12-week treatment. ** *P* < 0.01 versus SHAM group; ^#^ *P* < 0.05 versus OVX group; ^##^ *P* < 0.01 versus OVX group

Compared with the SHAM group, OVX caused remarkable atrophy of the uterus, indicating a successful operation. The administration of E2 or EXD remarkably relieved the atrophy of uterine tissue compared with the OVX rats (Fig. 2).

### Effect of EXD on the level of estradiol in serum

**Fig. 3:**
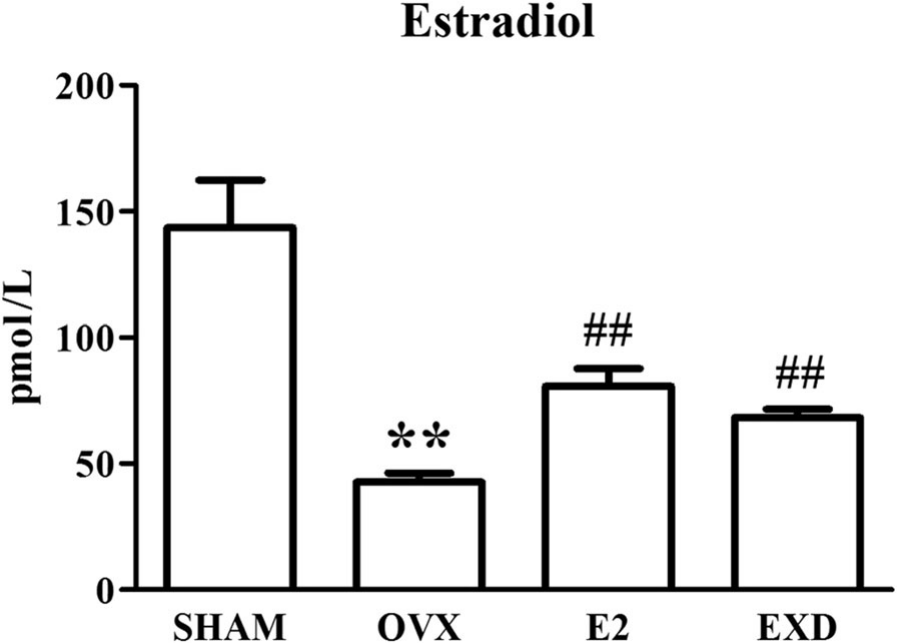
Effect of EXD on level of estradiol 2 in serum after 12-week treatment. ** *P* < 0.01 versus SHAM group; ^##^ *P* < 0.01 versus OVX group

Figure 3 shows the serum concentrations of estradiol in different rats after treatment for twelve weeks. After the 12-week treatment, the levels of estradiol in the SHAM rats were remarkably higher in comparison with the OVX rats (*p* < 0.01). In addition, the levels of estradiol in the EXD and E2 rats were remarkably lower in comparison with the OVX group rats.

### Effect of EXD on ventricular action potentials

Table 1 shows the effects of EXD on the ventricular action potential variables of model rats after treatment for twelve weeks. Compared with the SHAM group, OVX caused remarkable reductions in APD_50_ and APD_90_, and E2 or EXD could inhibit the reduction in APD_50_ significantly.

**Table 1:** Effect of EXD on ventricular action potentials

	RMP (mV)	Vmax (V/s)	APA (mV)	APD_50_ (ms)	APD_90_ (ms)
SHAM	−65.04 ± 6.01	10.05 ± 3.72	85.45 ± 17.04	50.32 ± 1.85	114.78 ± 16.03
OVX	−68.05 ± 4.51	10.98 ± 4.78	90.35 ± 10.12	34.75 ± 4.42^**^	89.14 ± 7.09^*^
E2	−60.88 ± 8.07	7.32 ± 2.24	82.59 ± 10.27	43.55 ± 3.07^#^	90.58 ± 11.87
EXD	−66.14 ± 9.20	8.08 ± 3.22	88.97 ± 13.54	44.00 ± 4.01^#^	95.59 ± 17.18

SHAM: sham-operated, OVX: ovariectomized, E2: 17β-estradiol, EXD: Er-Xian decoction, RMP: resting membrane potential, Vmax: maximal rate of depolarization, APA: AP amplitude, APD_50_: AP duration at 50%, APD_90_: AP duration at 90%. Values are presented as the means ± SD (*n* = 9 in each group). ^*^ *P* < 0.05 versus SHAM group; ^**^ *P* < 0.01 versus SHAM group; ^#^ *P* < 0.05 versus OVX group

### Effect of EXD on the left ventricle structure

**Fig. 4:**
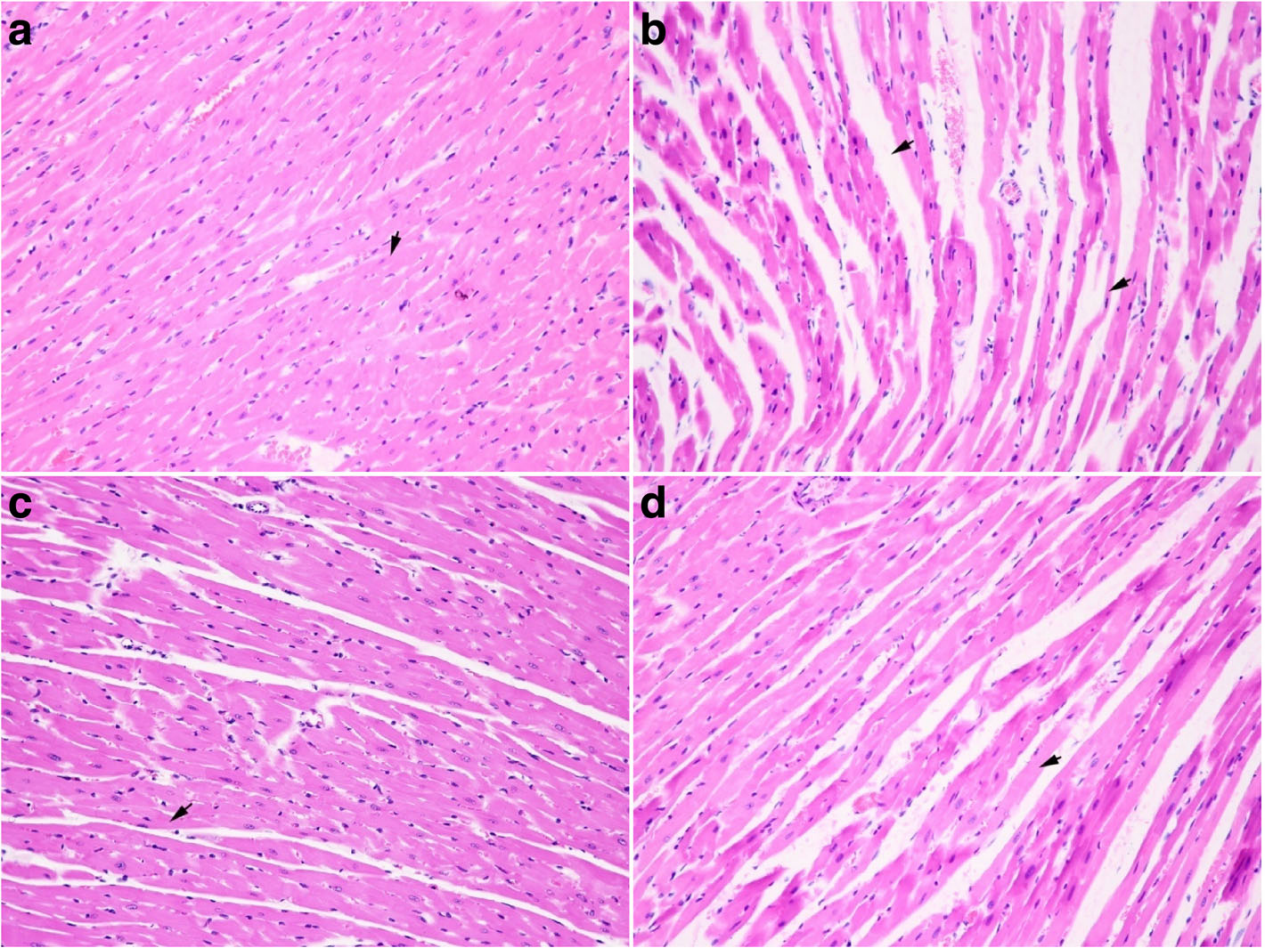
The effect of EXD on the left ventricle structure in OVX rats (HE staining, 20×). **a** Nornal myocardial fiber (indicated by arrowhead) in left ventricle of SHAM group rat; **b** Widening myocardial gap (indicated by arrowhead) and cytoplasmic condensation (indicated by arrowhead) shown in left ventricle of OVX group rat; **c** Myocardial fiber close to normal shown in left ventricle of E2 group rat; **d** Amelioration of cytoplasmic condensation in myocardial fiber shown in left ventricle of EXD group rat

A histopathological evaluation of ventricular samples from all groups was performed to explore the structural basis leading to the observed changes in APs. In the myocardium of SHAM rats, the cell alignment was normal and no obvious damage was detected (Fig. 4a). However, marked irregular cell arrangement, degeneration of ventricular myocytes, ruptured myocardial fibers, cytoplasmic vacuolization, and mild myocardial fibrosis were observed in OVX group rats (Fig. 4b). Upon E2 or EXD treatment, some mitigation of these events in the myocardial structure was observed in the rats (Fig. 4c-d).

### Effect of EXD on cardiomyocyte ultrastructural changes

**Fig. 5:**
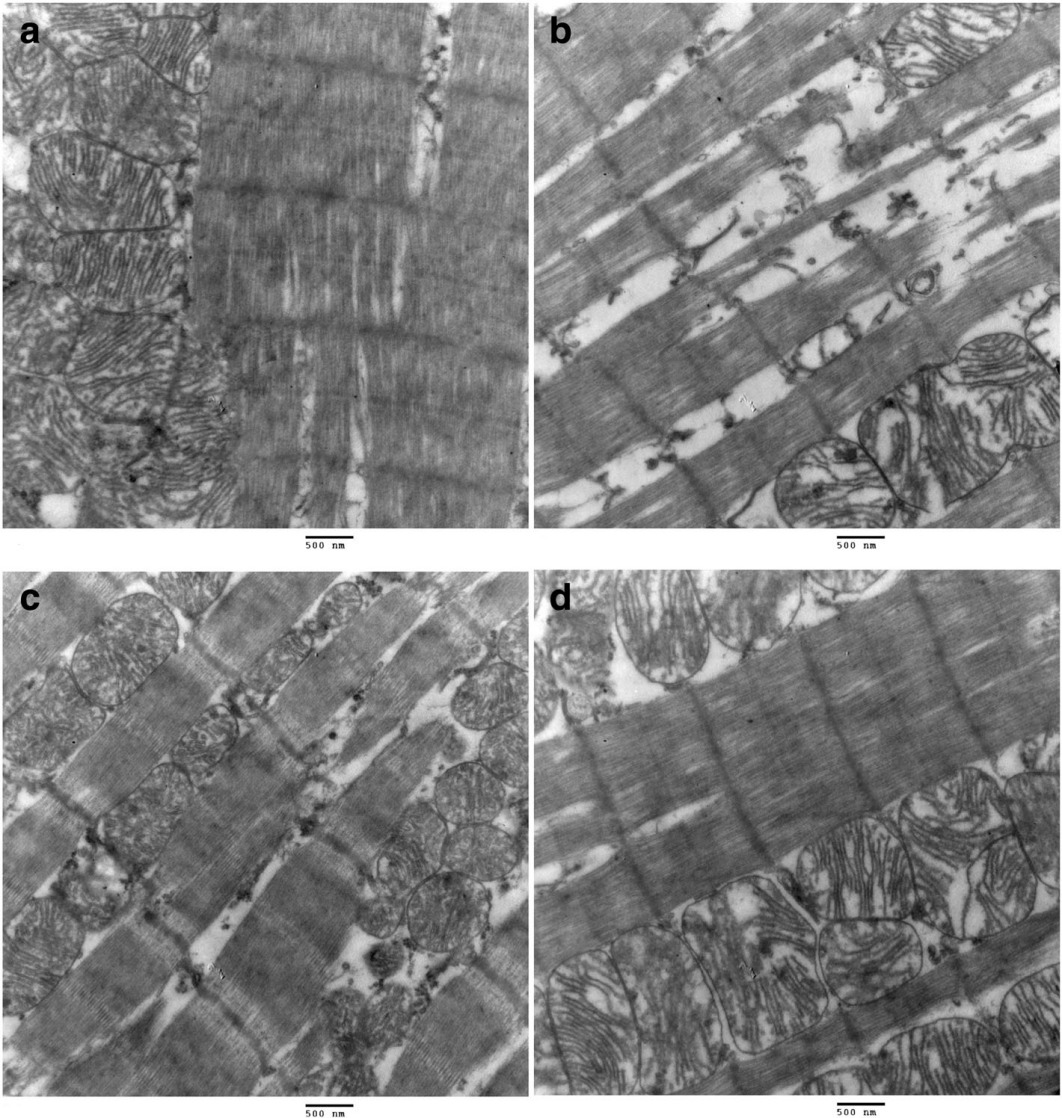
The effect of EXD on cardiomyocyte ultrastructural changes in rats (× 25,000). **a** SHAM; **b** OVX; **c**: E2; **d** EXD

The ultrastructures of cardiomyocytes from the SHAM rats demonstrated as normal (Fig. 5a), while the ultrastructures of cardiomyocytes from the OVX group rats demonstrated marked damages, which appeared as swollen and damaged mitochondria, intracellular edema, and myofibrillar derangements and ruptures (Fig. 5b). In contrast, significant decreases were observed in multiple characteristics of cardiomyocyte ultrastructural damages in the E2-or EXD-treated rats (Fig. 5c-d). All these features were ameliorated following EXD administration.

### Effect of EXD on gene expression profiles

The array data turned out that the expressions of 185 genes were changed (≥2-fold, *P* value≤0.05) between the ventricular muscles of the EXD and OVX rats. More precisely, 28 genes were up-regulated and 157 genes down-regulated (see Additional file 1).

### Pathway analysis of the differential expression genes

Based on the differential expression genes, we retrieved the top pathways (*P* value≤0.01) associated with myocardial morphology or function (Table 2) using the KEGG database. No pathway associated with down-regulated differentially expressed genes was found.

**Table 2:** Top pathways associated with down-regulated differentially expressed genes between the EXD group and OVX group

Pathways	*p* Value	FDR	Genes
Hypertrophic cardiomyopathy	5.47 × 10^−5^	0.005753	*Itgb5, Myh6, Myh7, Myl2, Myl3, Ttn*
Dilated cardiomyopathy	6.23 × 10^−5^	0.005753	*Itgb5, Myh6, Myh7, Myl2, Myl3, Ttn*
Cardiac muscle contraction	5.01 × 10^− 4^	0.034695	*Myh6, Myh7, Myl2, Myl3, Myl4*
Tight junction	7.56 × 10^−4^	0.041881	*Actn2, Ctnna3, Myh13, Myh6, Myh7, Myl2*
Arrhythmogenic right ventricular cardiomyopathy	2.70 × 10^−3^	0.124520	*Actn2, Ctnna3, Itgb5, Lef1*
Adrenergic signaling in cardiomyocytes	6.10 × 10^−3^	0.212027	*Myh6, Myh7, Myl2, Myl3, Myl4*
Viral myocarditis	6.12 × 10^−3^	0.212027	*Myh6, Myh7, RT1-CE7, RT1-T18*

### Confirmation of the differential expression genes by qRT-PCR

**Fig. 6:**
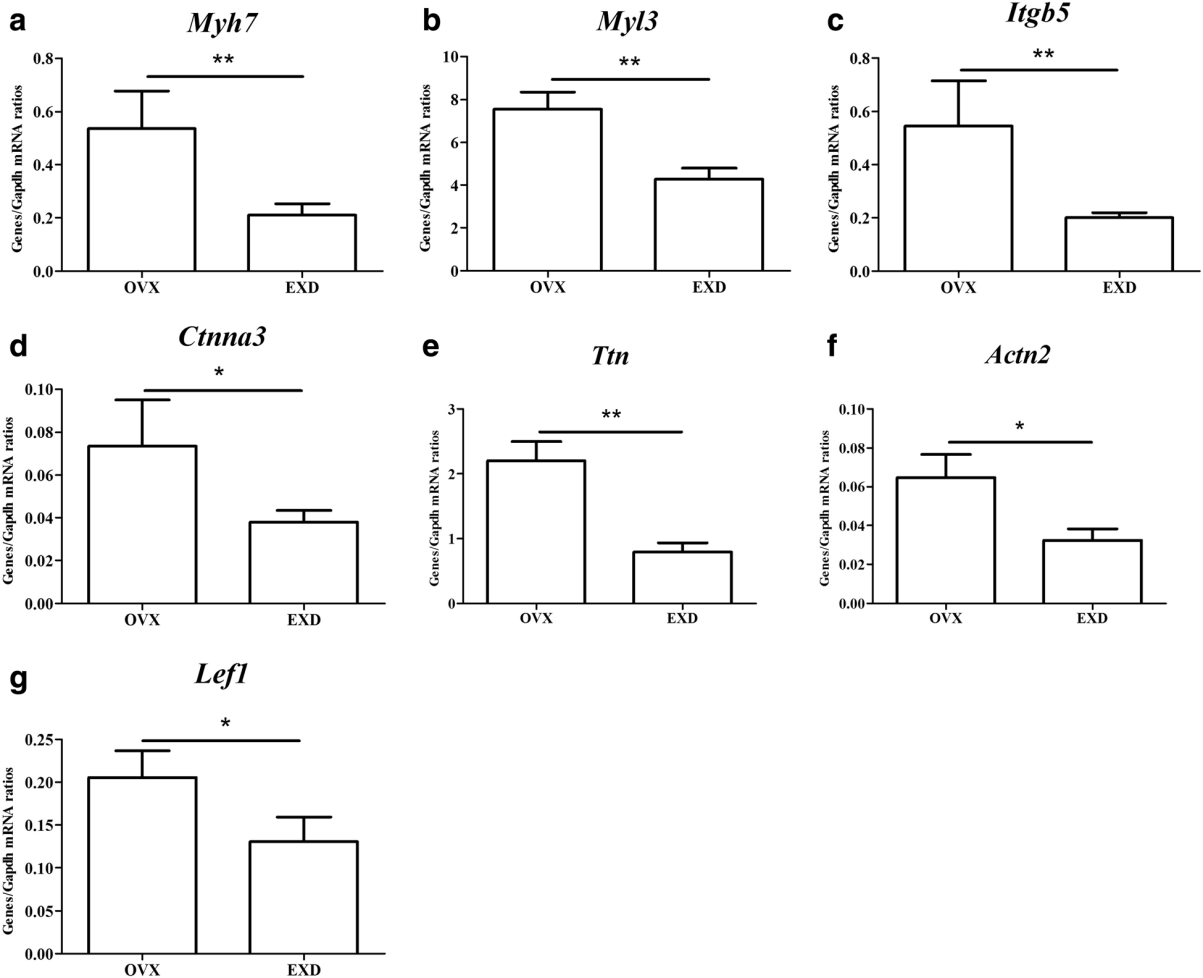
Validation of 7 differential expression genes (**a**: *Myh7*; **b**: *Myl3*; **c**: *Itgb5*; **d**: *Ctnna3*; **e**: *Ttn*; **f**: *Actn2*; **g**: *Lef1*) identified by microarray and KEGG in a replicated experiment by qRT-PCR. *P < 0.05, **P < 0.01, in comparison with the OVX group

To validate the differential expression genes, we chose 7 genes, including myosin light chain 3 (*Myl3*), myosin heavy chain 7 (*Myh7*), integrin subunit beta 5 (*Itgb5*), titin (*Ttn*), actinin alpha 2 (*Actn2*), catenin alpha 3 (*Ctnna3*), and lymphoid enhancer binding factor 1 (*Lef1*), which were involved in significant pathways in Table 2 and were associated with myocardial morphology or function. The primers of genes were listed in Table 3. The changes of these genes determined by qRT-PCR are shown in Fig. 6. In general, the results from qRT-PCR of all cases were consistent with the results identified in microarray analysis.

**Table 3:** Primers used for qRT-PCR analysis

Transcript	Sequence (5′-3′)
*Gapdh*	F:5’ GCTCTCTGCTCCTCCCTGTTCTA3’
	R:5’ TGGTAACCAGGCGTCCGATA3’
*Myh7*	F:5’TGCTGTTATTGCTGCCATTG3’
	R: 5’AGGAGTTATCATTCCGAACTGTC3’
*Myl3*	F:5’AAGAACAAAGACACGGGCAC3’
	R: 5’GTCAGCATTATGGTTGGGAGA3’
*Itgb5*	F:5’GAAAGCCCATCTCCACACATAC3’
	R: 5’CACTGAGCCATTGTAGGATTTGT3’
*Ctnna3*	F:5’AAGTCCGAAAAGAGAGCAAAGC3’
	R: 5’GCAAGAGGCACATGACATCAGT3’
*Ttn*	F:5’CCTGCGGTGGAAGGAGAATC3’
	R: 5’GGTTGTCAGGTCGTCCGTGT3’
*Actn2*	F:5’TTGCGACCAGTGGGATAGATT3’
	R: 5’TAGCAACTTCTCGGTTCTCTCC3’
*Lef1*	F:5’CAAGGTCAGCCTGTTTATCCC3’
	R: 5’GGAAGTGTCCCCTGAAAGTGA3’

## Discussion

In the current study, following ovariectomy, treatment with EXD or E2 for 12 weeks prevented the loss of uterine wet weight and body weight gain in OVX rats resulting from a lack of estrogen (Figs. 1 and 2). In addition, the results from the ECLIA assay showed that the serum estradiol levels of the EXD or E2 group rats were remarkably higher in comparison with the OVX group rats. We inferred that EXD has a slight estrogen-like effect that could slow down uterine atrophy and weight gain resulting from OVX.

Relationships were observed between contractility, action potentials and repolarization. During the plateau phase (phase 2) of the AP, characterized by a balance between inward calcium and outward potassium ions, a high concentration of intracellular calcium contributes to a cascade of actions that generate the mechanical contraction of myocardial cells [27].The outward potassium current (I_to_) can modulate contractility by regulating calcium ion transient amplitudes and kinetics [28]. From Table 1, we found that OVX could substantially shorten the ADP_50_ and ADP_90_ of model rats and that E2 or EXD could inhibit the reduction in APD_50_ in model rats after 12-week treatment. Some studies showed that E2 could prolong the APD of ventricular myocytes mainly by decreasing the rapidly activating component of the delayed I_to_ through estrogen receptor α [29, 30] and ameliorating the OVX-induced decrease in the cardiac contractile function of rats [31]. EXD showed a similar action to E2 on APD_50_ and we inferred that the estrogen-like activity of EXD plays an important role in this effect [32].

Changes in the histopathology of the left ventricle and in the ultrastructure of cardiomyocytes suggested that the cardiac tissue of OVX rats was impaired during estrogen deprivation, which is consistent with previous studies [33, 34]. E2 showed a cardioprotective effect and improved the cardiac structure in OVX rats as previously reported [35, 36]. Similar to E2, EXD could also mitigate myocardial injury in OVX rats after 12-week treatment. We speculated that the cardioprotective effect of EXD was associated with its estrogen-like activity.

The results from the electrophysiological and histomorphological observations demonstrated that EXD had a substantial myocardial protective effect similar to E2. To discover the mechanism of this cardioprotective effect of EXD, we screened the differential expression genes in the ventricular myocardium of the EXD group in comparison with the OVX group by microarray and identified key pathways with the KEGG. Based on the down-regulated differentially expressed genes, seven KEGG pathways associated with cardiomyopathy were retrieved (*P* value≤0.01, Table 2).

Sarcomeric proteins constitute approximately 80% of myofibril mass. The levels of multiple isoforms and post-translational modifications determine sarcomeric function. The sarcomeric proteins included several types, such as regulatory proteins (like troponins and tropomyosin), contractile proteins/myofilament proteins (like myosin and actin), and cytoskeletal proteins (like myosin binding protein C and titin). The force and motion generated by the myocardium come from myosin coupling its ATPase activity to its cyclic interaction with actin. Myosin consists of myosin heavy (Myh) chains and essential and regulatory myosin light (Myl) chains [37]. The cardiac myosin heavy chains of mammals possess two isoforms, the α-isoform and the β-isoform, which are encoded by two different genes, *Myh6* and *Myh7* [38]. More than ten myosin light chain isoforms have been discovered in skeletal muscle, smooth muscle and cardiac atrial muscle. *Myl2* is a regulatory myosin light chain and Myl3 and *Myl4* are essential myosin light chains [39].

In both menopausal women and in animal models, a lack of female hormones causes substantial myocardial injury [31, 40]. Many studies have indicated that female sex hormone deprivation induced by OVX shifted the expression of cardiac Myh chain isoforms from predominantly α-isoforms toward more β-isoforms [41, 42]. However, the expression of regulatory Myl chains in the myocardium of OVX mice or humans increased [43, 44]. Estrogen supplementation consistently abolished all these changes [45, 46]. In the present study, we found that EXD could decrease the expressions of *Myh7* and *Myl2* at the mRNA level in the ventricular myocardium, which could be one of the mechanisms of EXD inhibition of myocardial injury.

Integrins are a set of cell surface receptors involved in cellular adhesion and signaling. What’s more, they are considered as mechanotransducers in the circumstance of continuously contracting myocardium [47–49]. They have been associated with the progress of cardiac fibrosis or remodeling [50]. Cardiac fibroblasts can express αvβ5 integrin, which plays an importance part in cardiac remodeling via extracellular matrix-integrin interactions [51]. Our results from microarray and qRT-PCR demonstrated that the expression of *Itgb5* in EXD group rats significantly decreased in comparison with OVX group rats. We inferred that EXD may mitigate myocardial fibrosis in OVX rats by decreasing the expression of *Itgb5*.

Many studies proved that EXD could show estrogen-like effect [23, 32], and no toxicity about EXD was reported so far. In this research, we only explored the cardioprotective effect of EXD in an animal model. A line of studies had to be performed before EXD became a promising alternative preventive and therapeutic agent for myocardial injury in postmenopausal females.

## Conclusions

Based on the results from the electrophysiological test, histomorphological observations, microarray assay, pathway analysis and qRT-PCR validation, the current study demonstrated that treatment with EXD could lead to a cardioprotective effect against myocardial injury in OVX rats by modulating the expression of cardiac myosin or integrin at the mRNA level. Our study provided experimental evidence for EXD used as a drug for myocardial injury in postmenopausal females.

## Additional file

Additional file 1: Differentially expressed mRNA. This file listed 185 differentially expressed mRNA (≥2-fold, *P* value≤0.05) between the ventricular muscles of the EXD and OVX rats. (XLSX 17 kb)

## Abbreviations

AP: Action potential
APA: AP amplitude
APD50 and APD90: AP duration at 50% and 90%
ECLIA: Electrochemiluminescence immunoassay
EXD: Er-Xian decoction
HE: Hematoxylin and eosin
Ito: Outward potassium current
KEGG: Kyoto encyclopedia of genes and genomes
OVX: Ovariectomized
RMP: Resting membrane potential
TEM: Transmission electronmicroscope
Vmax: Maximal rate of depolarization
